# Crystal structures of three carbazole derivatives: 12-ethyl-7-phenyl­sulfonyl-7*H*-benzofuro[2,3-*b*]carbazole, (1), 2-(4,5-dimeth­oxy-2-nitro­phen­yl)-4-hy­droxy-9-phenyl­sulfonyl-9*H*-carbazole-3-carbaldehyde, (2), and 12-phenyl-7-phenyl­sulfonyl-7*H*-benzofuro[2,3-*b*]carbazole, (3)

**DOI:** 10.1107/S2056989016016819

**Published:** 2016-11-04

**Authors:** Rajeswari Gangadharan, P. Narayanan, K. Sethusankar, Velu Saravanan, Arasambattu K. Mohanakrishnan

**Affiliations:** a Department of Physics, Ethiraj College for Women (Autonomous), Chennai 600 008, India; b Department of Physics, RKM Vivekananda College (Autonomous), Chennai 600 004, India; c Department of Organic Chemistry, University of Madras, Guindy Campus, Chennai 600 025, India

**Keywords:** crystal structure, benzo­furan, carbazole derivatives, inversion dimers, aggregation, C—H⋯O hydrogen bonds, C—H⋯π inter­actions

## Abstract

The three title compounds, C_26_H_19_NO_3_S, (1), C_27_H_20_N_2_O_8_S, (2), and C_30_H_19_NO_3_S, (3), are carbazole derivatives, where (1) and (3) are heterocycle-containing carbazoles with a benzo­furan moiety fused to a carbazole unit. In (2), a di­meth­oxy­nitro­phenyl ring is attached to the carbazole moiety. In the three derivatives, a phenyl­sulfonyl group is attached to the N atom of the carbazole unit. Compound (1) crystallizes with two independent mol­ecules in the asymmetric unit (*A* and *B*). The carbazole skeleton in the three compounds is essentially planar. In compound (1), the benzene ring of the phenyl­sulfonyl moiety is almost orthogonal to the carbazole moiety, with dihedral angles of 85.42 (9) and 84.52 (9)° in mol­ecules *A* and *B*, respectively. The benzene ring of the phenyl­sulfonyl group in compounds (2) and (3) are inclined to the carbazole moiety, making dihedral angles of 70.73 (13) and 81.73 (12)°, respectively. The S atom has a distorted tetra­hedral configuration in all three compounds. In the crystals, C—H⋯O hydrogen bonds give rise to *R*_2_^2^(12) inversion dimers for compound (1), and to *R*_2_^2^(24) inversion dimers and *R*_4_^4^(40) ring motifs for compound (2). The crystal packing in (1) also features C—H⋯π and π–π inter­actions [shortest inter­centroid distance = 3.684 (1) Å], leading to supra­molecular three-dimensional aggregation. In the crystal of compound (2), the combination of the various C—H⋯O hydrogen bonds leads to the formation of a three-dimensional network. In the crystal of compound (3), mol­ecules are linked by C—H⋯O hydrogen bonds, forming chains running parallel to the *a* axis, and the chains are linked by C—H⋯π inter­actions, forming corrugated sheets parallel to the *ab* plane.

## Abstract 2

The title compounds are carbazole derivatives. In all three compounds, the carbazole skeleton is essentially planar. In two of the compounds, a benzo­furan moiety is fused with a carbazole unit. Inter­molecular C—H⋯O hydrogen bonds give rise to 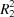
(12) inversion dimers in one compound, and to 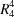
(40) ring motifs and 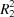
(24) inversion dimers in a second compound. In the crystal of the third compound, C—H⋯O hydrogen bonds link the mol­ecules to form chains running parallel to the *a* axis.

## Chemical context   

Carbazoles are widely used as building blocks for new organic materials and play an important role in electroactive and photoactive devices. Carbazole derivatives have also been used as luminescent and hole-transporting materials (Dijken *et al.*, 2004 ▸). These compounds are also thermally and phytochemically stable which makes them useful materials for technological applications (Diaz *et al.*, 2002 ▸).

Heterocycle-containing carbazole derivatives are embodied in many natural products (Itoigawa *et al.*, 2000 ▸) and display a broad spectrum of useful biological activities, such as anti­tumour, anti­mitotic and anti­oxidative activities (Prudhomme, 2003 ▸; Tachibana *et al.*, 2003 ▸; Hu *et al.*, 2006 ▸). A number of benzo-annulated carbazole ring systems containing an aromatic ring fused to the carbazole nucleus are potential candidates for cancer treatment as a result of their DNA inter­calative binding properties. They have been shown to bind to estrogen receptors and exhibit a pronounced anti­tumor activity against leukemia, renal tumor, colon cancer and malignant melanoma tumor cell lines (Pindur & Lemster, 1997 ▸).
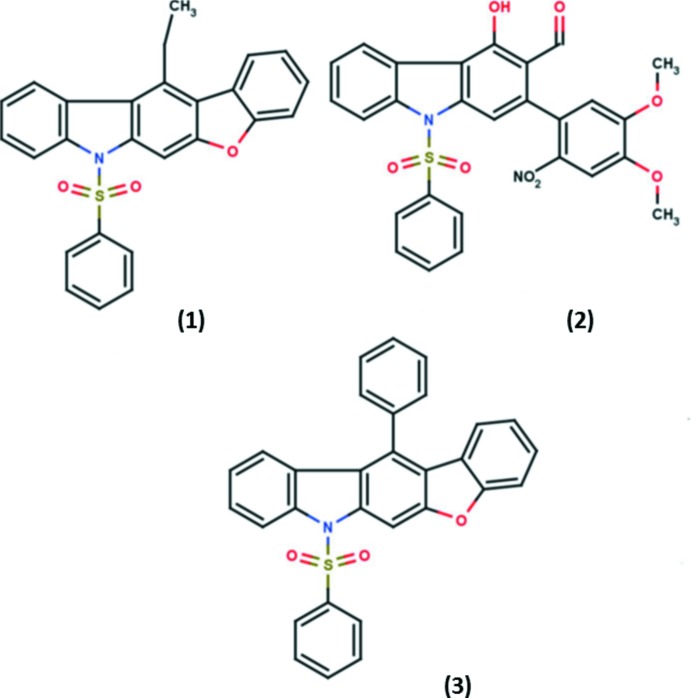


Most heterocycle-containing carbazoles reported in the literature comprise a common heterocyclic ring moiety fused with a carbazole ring system, such as pyridocarbazoles and indolocarbazoles. In this context, we discuss here three carbazole derivatives, two of which have benzo­furan moieties fused with the carbazole unit.

## Structural commentary   

**Figure 1:**
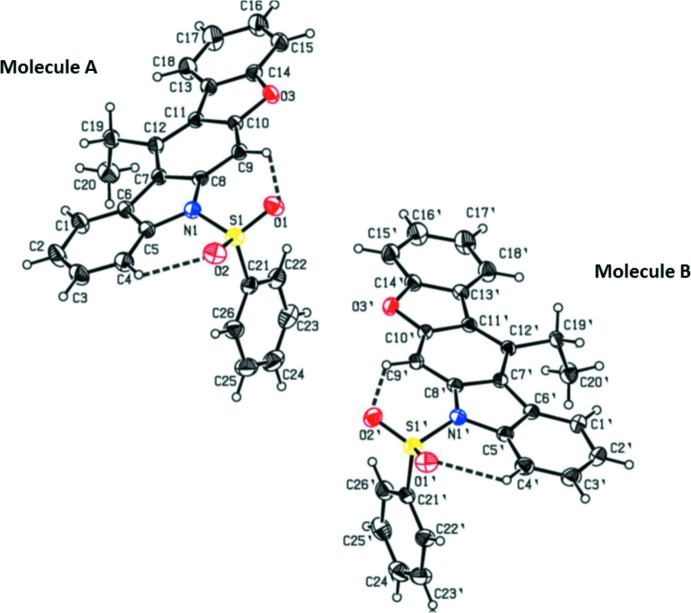
The mol­ecular structure of compound (1), showing the atom-numbering scheme and displacement ellipsoids drawn at the 30% probability level. The intra­molecular C—H⋯O hydrogen bonds, which generate two *S*(6) ring motifs, are shown as dashed lines (see Table 1 ▸).

**Figure 2:**
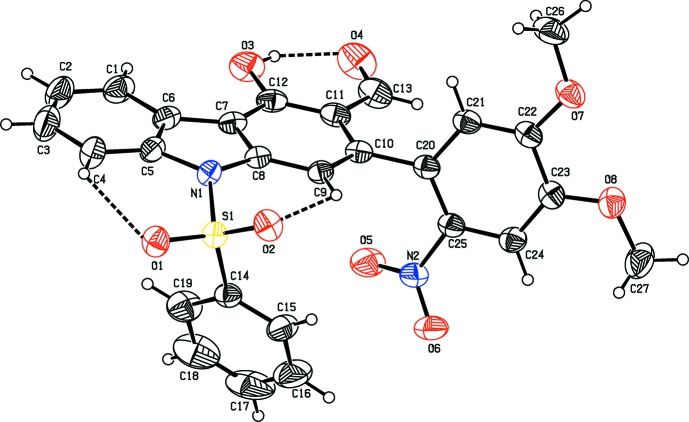
The mol­ecular structure of compound (2), showing the atom-numbering scheme and displacement ellipsoids are drawn at the 30% probability level. The intra­molecular O—H⋯O and C—H⋯O hydrogen bonds, which generate three *S*(6) ring motifs, are shown as dashed lines (see Table 2 ▸).

The three title compounds C_26_H_19_NO_3_S, (1), C_27_H_20_N_2_O_8_S, (2), and C_30_H_19_NO_3_S, (3), are carbazole derivatives, where (1) and (3) are heterocycle-containing carbazoles with a benzo­furan fused to the carbazole skeleton (Figs. 1 ▸ and 3 ▸, respectively). In (2), a di­meth­oxy­nitro­phenyl ring is attached to the carbazole moiety (Fig. 2 ▸). In the three derivatives, a phenyl­sulfonyl group is attached to the N atom of the carbazole unit. Compound (1) crystallizes with two independent mol­ecules (*A* and *B*) in the asymmetric unit, as shown in Fig. 1 ▸. The carbazole skeleton in the three compounds is essentially planar [maximum deviations of 0.052 (2) Å for atom C12 in mol­ecule *A* and 0.080 (2) Å for atom C12′ in mol­ecule *B* of (1), −0.034 (2) Å for atom C10 in (2), and −0.042 (4) Å for atom C3 in (3)]. The carbazole benzo­furan fused penta­cyclic unit is almost planar in (1) and (3), with dihedral angles between the benzo­furan and carbazole units being 2.48 (6) and 4.16 (6)° in mol­ecules *A* and *B*, respectively of (1), and 2.33 (8)° in compound (3). In compound (1), the benzene ring of the phenyl­sulfonyl group is almost orthogonal to the carbazole moiety, with the dihedral angles between their mean planes being 85.42 (9) and 84.52 (9)° in mol­ecules *A* and *B*, respectively. The benzene ring of the phenyl­sulfonyl group in compounds (2) and (3) are inclined to the carbazole moiety making dihedral angles of 70.73 (12) and 81.73 (12)°, respectively.

In all three compounds, there are two intra­molecular C—H⋯O hydrogen bonds, involving the sulfonyl ring O atoms forming two cyclic *S*(6) motifs (Tables 1 ▸, 2 ▸ and 3 ▸). In compound (2), an O—H⋯O hydrogen bond generates an additional *S*(6) ring motif (Table 2 ▸). Atom S1 has a distorted tetra­hedral geometry in all three compounds. The widening of angle O2=S1=O1 [119.55 (10) and 119.46 (10)° in mol­ecules *A* and *B*, respectively, of (1), 119.78 (10)° in (2) and 119.99 (13)° in (3)] and narrowing of angle N—S—C [104.85 (9) and 104.82 (9)° in mol­ecules *A* and *B*, respectively, of (1), 102.92 (9)° in (2) and 105.79 (12)° in (3)] from the ideal tetra­hedral value are attributed to the Thorpe–Ingold effect (Bassindale, 1984 ▸). As a result of the electron-withdrawing character of the phenyl­sulfonyl group, the bond lengths N1—C5 [1.430 (2) and 1.431 (2) Å in mol­ecules *A* and *B* of (1), 1.429 (3) Å in (2) and 1.432 (4) Å in (3)] and N1—C8 [1.428 (2) and 1.425 (2) Å in mol­ecules *A* and *B* of (1), 1.414 (2) Å in (2) and 1.432 (3) Å in (3)] in all three compounds are longer than the normal value of 1.355 (14) Å [Cambridge Structural Database (CSD), Version 5.37; last update May 2016,; Groom *et al.*, 2016 ▸].

In compound (2), the di­meth­oxy­nitro­phenyl ring makes a dihedral angle of 76.63 (8)° with the carbazole moiety. The nitro group in (2) is (+) syn-periplanar to the phenyl ring (atoms C20–C25), as indicated by the values of the torsion angles C24—C25—N2—O6 = 21.4 (3)° and C20—C25—N2—O5 = 19.9 (3)°. The torsion angles C22—C23—O8—C27 = −174.6 (2)° and C23—C22—O7—C26 = 175.9 (2)° indicate that the two meth­oxy substituents at C23 and C22 are almost coplanar with the phenyl ring.

In compound (3), the phenyl ring attached at C12 is oriented at a dihedral angle of 78.39 (11)° to the carbazole unit.

## Supra­molecular features   

**Figure 4:**
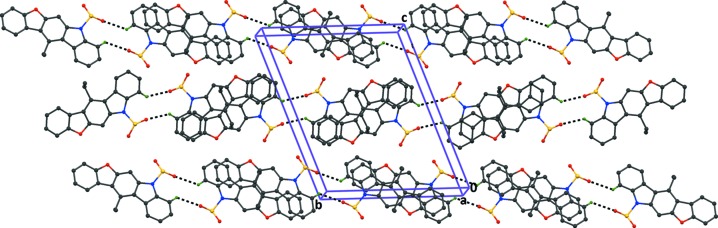
The crystal packing of compound (1), viewed along the *a*-axis, showing the formation of centrosymmetric A—A dimers, with descriptor 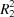
(12). The dashed lines indicate the inter­molecular C—H⋯O hydrogen bonds (Table 1 ▸) and H atoms not involved in hydrogen bonding, and the phenyl ring of the phenyl­sulfonate groups, have been excluded for clarity.

In the crystal of compound (1), mol­ecules are linked *via* C4—H4⋯O2 and C4′—H4′⋯O1′ hydrogen bonds, generating two 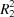
(12) inversion dimers (Table 1 ▸ and Fig. 4 ▸). The crystal packing also features C—H⋯π (Table 1 ▸) and π–π inter­actions leading to supra­molecular three-dimensional aggregation. The π–π inter­actions involve inversion related *A* mol­ecules with an inter­centroid distance *Cg*4⋯*Cg*4^i^ = 3.703 (2) Å [where *Cg*4 is the centroid of ring C7–C12; symmetry code: (i) −*x* + 2, −*y* + 1, −*z* + 1], and inversion related *B* mol­ecules, with an inter­centroid distance *Cg*20⋯*Cg*20^ii^ = 3.684 (2) Å [where *Cg*20 is the centroid of ring C7′–C12′; symmetry code: (ii) −*x* + 1, −*y* + 1, −*z*].

**Figure 5:**
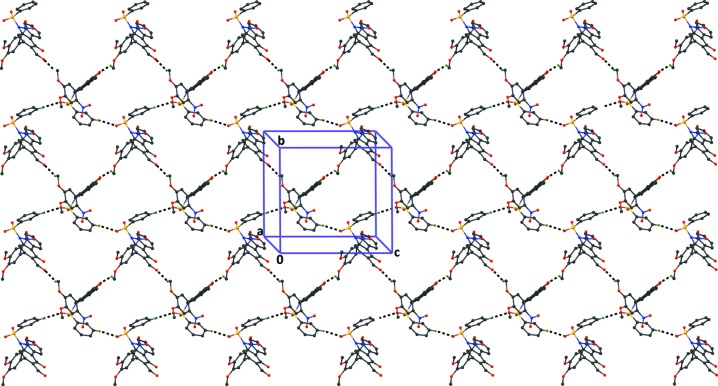
The crystal packing of compound (2), viewed along the *a* axis, showing the formation of 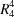
(40) graph-set ring motifs, resulting in the formation of sheets parallel to the *bc* plane. The dashed lines indicate the C—H⋯O hydrogen bonds (Table 2 ▸), and H atoms not involved in the hydrogen bonding have been excluded for clarity.

**Figure 6:**
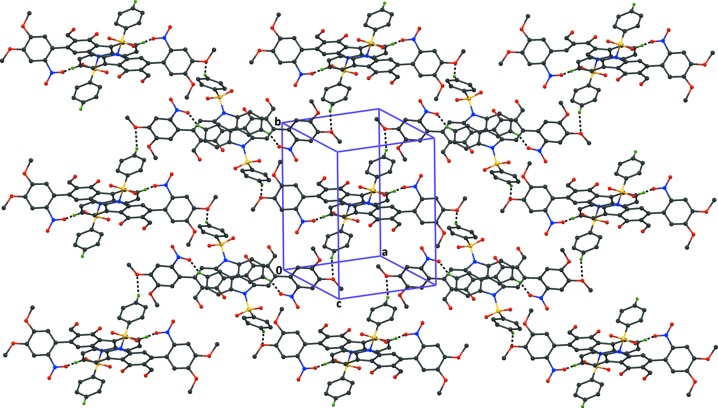
The crystal packing of compound (2), viewed normal to plane (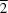
04), showing the formation of 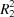
(24) graph-set ring motifs, resulting in the formation of sheets parallel to plane (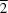
04). The dashed lines indicate the inter­molecular C—H⋯O hydrogen bonds (Table 2 ▸), and H atoms not involved in the hydrogen bonding have been excluded for clarity.

In the crystal of compound (2), neighbouring mol­ecules are linked by C18—H18⋯O2^iii^ and C26—H26*C*⋯O4^iv^ hydrogen bonds forming 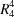
(40) ring motifs resulting in the formation of sheets parallel to the *bc* plane (Table 2 ▸ and Fig. 5 ▸). Mol­ecules are also linked *via* C2—H2⋯O5^i^ hydrogen bonds which form 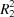
(24) inversion dimers. These dimers are further crosslinked by C17—H17⋯O8^ii^ hydrogen bonds (Table 2 ▸), forming sheets parallel to plane (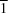
02); as shown in Fig. 6 ▸. The sum of these inter­actions is the formation of a three-dimensional hydrogen-bonded framework.

**Figure 7:**
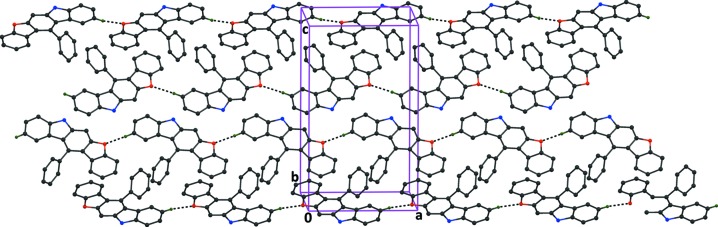
The crystal packing of compound (3), viewed along the *b* axis, showing the C—H⋯O hydrogen bonds (dashed lines; Table 3 ▸), which generate *C(9)* chains running parallel to the *a* axis. H atoms not involved in the hydrogen bonding, and the phenyl­sulfonate groups, have been excluded for clarity.

In the crystal of compound (3), mol­ecules are linked through C2—H2⋯O3^i^ hydrogen bonds (Table 3 ▸), that generate infinite one-dimensional *C*(9) chains running parallel to the *a* axis (Fig. 7 ▸). The chains are further crosslinked by C17—H17⋯*Cg*4^ii^ and C22—H22⋯*Cg*3^iii^ inter­actions (Table 3 ▸), which results in the formation corrugated sheets parallel to the *ab* plane.

## Database survey   

A search of the CSD (Groom *et al.*, 2016 ▸) revealed two closely related structures including the parent compound 7*H*-1-benzofuro[2,3-*b*]carbazole (Panchatcharam *et al.*, 2011*a* ▸). This carbazole–benzo­furan fused penta­cyclic unit crystallizes in the space group *Pca*2_1_. However, compound 7-phenyl­sufonyl-7*H*-benzo­furan­[2,3-*b*]carbazole (Panchatcharam *et al.*, 2011*b* ▸) is the closest analogue to the title compounds (1) and (3), and crystallizes in the space group *P*2_1_/*c*. The presence of an ethyl or phenyl substituent attached to the carbazole unit does not cause much variation in the structural parameters. The packing of the title compounds are consolidated by C–H⋯O inter­actions, but the related compounds exhibit only C—H⋯π and π–π inter­actions.

A similar search conducted for compound (2) gave 10 hits of compounds having a phenyl ring attached to a 7-phenyl­sulfonyl-7*H*-benzo­furan­[2,3-*b*]carbazole skeleton. The closest analogues to compound (2) are 2-(4,5-dimeth­oxy-2-nitro­phen­yl)-4-meth­oxy-3-methyl-9-phenyl­sulfonyl-9*H*-carbazole (Narayanan *et al.*, 2014*a* ▸) and 2-(4,5-dimeth­oxy-2-nitro­phen­yl)-4-meth­oxy-9-phenyl­sufonyl-9*H*carbazole-3-carbaldehyde (Narayanan *et al.*, 2014*b* ▸). Both crystallize in the space group *Pca*2_1_, and differ from compound (2) only in the groups attached to the substituted phenyl ring of the carbazole moiety.

## Synthesis and crystallization   

For the preparation of compound (1), a solution of [1-(phenyl­sulfon­yl)-3-propionyl-1H-indol-2-yl]methyl pivalate (0.1 g, 2.34 mmol), anhydrous SnCl_4_ (0.07 g, 2.81 mmol) and benzo­furan (0.033 g, 2.81 mmol) in dry DCE (10 ml) was stirred at room temperature under a nitro­gen atmosphere for 3 h. After the completion of the reaction (monitored by thin-layer chromatography, TLC), it was poured into ice water (100 ml), the organic layer was separated and the aqueous layer was extracted with DCM (2 × 20 ml). The combined extract was washed with water (3 × 50 ml) and dried (Na_2_SO_4_). Removal of solvent followed by column chromatographic purification (silica gel; hexa­ne–ethyl acetate, 8:2 *v*/*v*) led to the isolation of compound (1) as a colourless solid (yield 0.064 g, 64%; m.p. 483–485 K).

For the preparation of compound (2), to a solution of 4-meth­oxy­carbazole-3-carbaldehydes (0.82 g, 1.5 mmol) in dry DCM (20 ml), 1 *M* solution of BBr_3_ (1.65 ml, 1.65 mmol) in DCM was added at 273 K. After completion of the reaction (monitored by TLC), it was poured into ice water (50 ml) containing HCl (5 ml). The organic layer was separated and the aqueous layer was then extracted with DCM (2 × 10 ml). The combined organic layer was washed water (2 × 30 ml) and dried (Na_2_SO_4_). Removal of the solvent followed by trituration of the crude product with MeOH (10 ml) gave compound (2) as a pale-yellow solid (yield 0.73 g, 92%; m.p. 467–469 K).

For the preparation of compound (3), a solution of [3-benzoyl-1-(phenyl­sulfon­yl)-1*H*-indol-2-yl]methyl pivalate (0.1 g, 2.11 mmol), anhydrous SnCl_4_ (0.066 g, 2.52 mmol) and benzo­furan (0.03 g, 2.52 mmol) in dry DCE (10 ml) was stirred at room temperature under a nitro­gen atmosphere for 3 h. After the completion of the reaction (monitored by TLC), it was poured into ice water (100 ml), the organic layer was separated and the aqueous layer was extracted with DCM (2 × 20 ml). The combined extract was washed with water (3 × 50 ml) and dried (Na_2_SO_4_). Removal of solvent followed by column chromatographic purification (silica gel; hexa­ne–ethyl acetate, 8:2 *v*/*v*) gave compound (3) as a colourless solid (yield 0.07 g, 70%; m.p. 491–493 K). Colourless block-like crystals were obtained by slow evaporation of a solution of (1) and (3) in ethyl acetate. Yellow block-like crystals were obtained by slow evaporation of a solution of (2) in methanol.

## Refinement   

Crystal data, data collection and structure refinement details for compounds (1), (2) and (3) are summarized in Table 4 ▸. The H atoms were included in calculated positions and treated as riding atoms: O—H = 0.82 Å, C—H = 0.93–0.97 Å, with *U*_iso_(H)= 1.5*U*_eq_(hy­droxy O and methyl C) and 1.2*U*_eq_(C) for other H atoms. The methyl groups were allowed to rotate, but not to tip, to best fit the electron density.

## Supplementary Material

Crystal structure: contains datablock(s) 1, 2, 3, global. DOI: 10.1107/S2056989016016819/su5330sup1.cif

Supporting information file. DOI: 10.1107/S2056989016016819/su53301sup2.cml

Supporting information file. DOI: 10.1107/S2056989016016819/su53302sup3.cml

Supporting information file. DOI: 10.1107/S2056989016016819/su53303sup4.cml

CCDC references: 1479200, 1479199, 1479198

Additional supporting information: crystallographic information; 3D view; checkCIF report

## Figures and Tables

**Figure 3:**
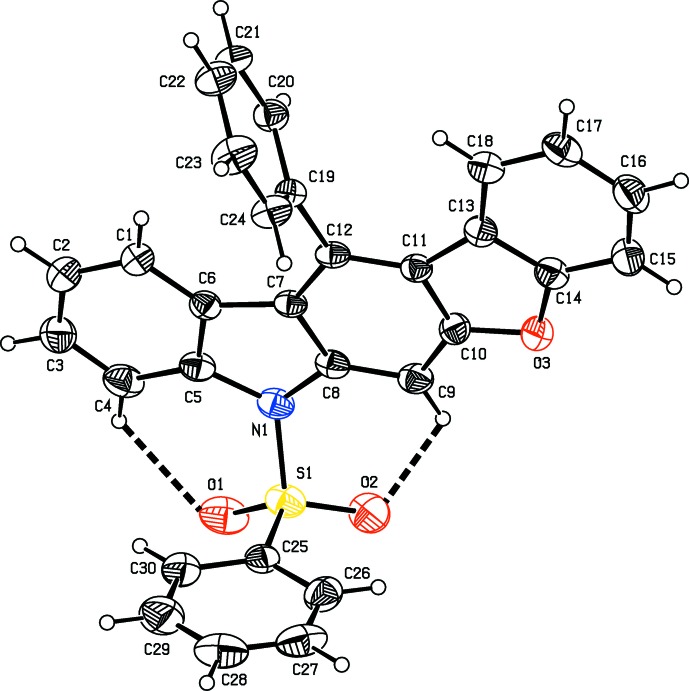
The mol­ecular structure of compound (3), showing the atom-numbering scheme and displacement ellipsoids drawn at the 30% probability level. The intra­molecular C—H⋯O hydrogen bonds, which generate two *S*(6) ring motifs, are shown as dashed lines (see Table 3 ▸).

**Table 1:** Hydrogen-bond geometry (Å, °) for (1) *Cg*1, *Cg*4, *Cg*6, *Cg*17 and *Cg*20 are the centoids of rings O3/C10/C11/C13/C14, C7–C12, C21–C26, O3′/C10′/C11′/C13′/C14′ and C7′–C12′, respectively.

*D*—H⋯*A*	*D*—H	H⋯*A*	*D*⋯*A*	*D*—H⋯*A*
C4—H4⋯O2	0.93	2.35	2.954 (3)	122
C4′—H4′⋯O1′	0.93	2.36	2.966 (3)	122
C9—H9⋯O1	0.93	2.29	2.881 (2)	121
C9′—H9′⋯O2′	0.93	2.28	2.875 (2)	121
C4—H4⋯O2^i^	0.93	2.53	3.277 (3)	137
C20′—H20*A*⋯*Cg*17^ii^	0.96	2.82	3.449 (3)	124
C20′—H20*C*⋯*Cg*20^ii^	0.96	2.79	3.427 (3)	125
C20—H20*D*⋯*Cg*4^iii^	0.96	2.83	3.464 (3)	124
C20′—H20*C*⋯*Cg*1^iii^	0.96	2.85	3.478 (3)	124
C25′—H25′⋯*Cg*6^iv^	0.93	2.90	3.762 (3)	155

Symmetry codes: (i) 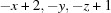; (ii) 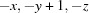; (iii) 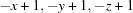; (iv) 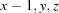.

**Table 2:** Hydrogen-bond geometry (Å, °) for (2)

*D*—H⋯*A*	*D*—H	H⋯*A*	*D*⋯*A*	*D*—H⋯*A*
O3—H3*A*⋯O4	0.82	1.83	2.554 (3)	146
C4—H4⋯O1	0.93	2.29	2.866 (3)	119
C9—H9⋯O2	0.93	2.47	3.054 (2)	121
C2—H2⋯O5^i^	0.93	2.50	3.281 (3)	142
C17—H17⋯O8^ii^	0.93	2.59	3.481 (5)	161
C18—H18⋯O2^iii^	0.93	2.51	3.384 (5)	157
C26—H26*C*⋯O4^iv^	0.96	2.50	3.265 (4)	137

Symmetry codes: (i) 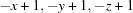; (ii) 
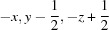
; (iii) 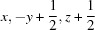; (iv) 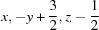.

**Table 3:** Hydrogen-bond geometry (Å, °) for (3) *Cg*3 and *Cg*4 are the centroids of rings C1–C6 and C7–C12, respectively.

*D*—H⋯*A*	*D*—H	H⋯*A*	*D*⋯*A*	*D*—H⋯*A*
C4—H4⋯O1	0.93	2.34	2.924 (4)	121
C9—H9⋯O2	0.93	2.34	2.926 (3)	121
C2—H2⋯O3^i^	0.93	2.57	3.464 (4)	160
C17—H17⋯*Cg*4^ii^	0.93	2.81	3.683 (3)	156
C22—H22⋯*Cg*3^iii^	0.93	2.95	3.722 (3)	141

Symmetry codes: (i) 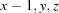; (ii) 
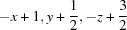
; (iii) 
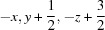
.

**Table 4:** Experimental details

	(1)	(2)	(3)
Crystal data			
Chemical formula	C_26_H_19_NO_3_S	C_27_H_20_N_2_O_8_S	C_30_H_19_NO_3_S
*M*_r_	425.48	532.51	473.52
Crystal system, space group	Triclinic, *P* 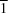	Monoclinic, *P*2_1_/*c*	Orthorhombic, *P*2_1_2_1_2_1_
Temperature (K)	296	296	296
*a*, *b*, *c* (Å)	8.3037 (2), 14.3468 (3), 18.4068 (5)	11.2133 (3), 14.5811 (4), 15.1509 (4)	10.6461 (10), 11.8994 (11), 18.2418 (16)
α, β, γ (°)	70.594 (1), 78.139 (1), 85.356 (1)	90, 102.320 (1), 90	90, 90, 90
*V* (Å^3^)	2023.90 (8)	2420.16 (11)	2310.9 (4)
*Z*	4	4	4
Radiation type	Mo *K*α	Mo *K*α	Mo *K*α
μ (mm^−1^)	0.19	0.19	0.17
Crystal size (mm)	0.35 × 0.30 × 0.25	0.35 × 0.30 × 0.25	0.35 × 0.30 × 0.25

Data collection			
Diffractometer	Bruker Kappa APEXII CCD	Bruker Kappa APEXII CCD	Bruker Kappa APEXII CCD
Absorption correction	Multi-scan (*SADABS*; Bruker, 2008 ▸)	Multi-scan (*SADABS*; Bruker, 2008 ▸)	Multi-scan (*SADABS*; Bruker, 2008 ▸)
*T*_min_, *T*_max_	0.936, 0.954	0.935, 0.953	0.941, 0.957
No. of measured, independent and observed [*I* > 2σ(*I*)] reflections	32388, 8968, 7032	42635, 6535, 4517	29269, 5055, 3194
*R*_int_	0.022	0.025	0.061
(sin θ/λ)_max_ (Å^−1^)	0.643	0.690	0.640

Refinement			
*R*[*F* ^2^ > 2σ(*F* ^2^)], *wR*(*F* ^2^), *S*	0.046, 0.125, 1.01	0.047, 0.150, 1.00	0.042, 0.101, 1.00
No. of reflections	8968	6535	5055
No. of parameters	561	345	316
No. of restraints	0	0	1
H-atom treatment	H-atom parameters constrained	H-atom parameters constrained	H-atom parameters constrained
Δρ_max_, Δρ_min_ (e Å^−3^)	0.67, −0.37	0.33, −0.46	0.16, −0.25
Absolute structure	–	–	Flack (1983 ▸), 2189 Friedel pairs
Absolute structure parameter	–	–	0.08 (9)

Computer programs: *APEX2* (Bruker, 2008 ▸), *SAINT* (Bruker, 2008 ▸), *SHELXS97* (Sheldrick, 2008 ▸), *ORTEP-3 for Windows* (Farrugia, 2012 ▸) and *Mercury* (Macrae *et al.*, 2008 ▸), *SHELXL97* (Sheldrick, 2008 ▸) and *PLATON* (Spek, 2009 ▸).

## supplementary crystallographic information

### Crystal data

**Table:**

C_30_H_19_NO_3_S	*F*(000) = 984
*M**_r_* = 473.52	*D*_x_ = 1.361 Mg m^−^^3^
Orthorhombic, *P*2_1_2_1_2_1_	Mo *K*α radiation, λ = 0.71073 Å
Hall symbol: P 2ac 2ab	Cell parameters from 5055 reflections
*a* = 10.6461 (10) Å	θ = 2.2–27.1°
*b* = 11.8994 (11) Å	µ = 0.17 mm^−^^1^
*c* = 18.2418 (16) Å	*T* = 296 K
*V* = 2310.9 (4) Å^3^	Block, colourless
*Z* = 4	0.35 × 0.30 × 0.25 mm

### Data collection

**Table:**

Bruker Kappa APEXII CCD diffractometer	5055 independent reflections
Radiation source: fine-focus sealed tube	3194 reflections with *I* > 2σ(*I*)
Graphite monochromator	*R*_int_ = 0.061
ω & φ scans	θ_max_ = 27.1°, θ_min_ = 2.2°
Absorption correction: multi-scan (SADABS; Bruker, 2008)	*h* = −13→13
*T*_min_ = 0.941, *T*_max_ = 0.957	*k* = −15→15
29269 measured reflections	*l* = −23→22

### Refinement

**Table:**

Refinement on *F*^2^	Secondary atom site location: difference Fourier map
Least-squares matrix: full	Hydrogen site location: inferred from neighbouring sites
*R*[*F*^2^ > 2σ(*F*^2^)] = 0.042	H-atom parameters constrained
*wR*(*F*^2^) = 0.101	*w* = 1/[σ^2^(*F*_o_^2^) + (0.0488*P*)^2^] where *P* = (*F*_o_^2^ + 2*F*_c_^2^)/3
*S* = 1.00	(Δ/σ)_max_ < 0.001
5055 reflections	Δρ_max_ = 0.16 e Å^−^^3^
316 parameters	Δρ_min_ = −0.25 e Å^−^^3^
1 restraint	Absolute structure: Flack (1983), 2189 Friedel pairs
Primary atom site location: structure-invariant direct methods	Absolute structure parameter: 0.08 (9)

### Special details

**Table:**

Geometry. All esds (except the esd in the dihedral angle between two l.s. planes) are estimated using the full covariance matrix. The cell esds are taken into account individually in the estimation of esds in distances, angles and torsion angles; correlations between esds in cell parameters are only used when they are defined by crystal symmetry. An approximate (isotropic) treatment of cell esds is used for estimating esds involving l.s. planes.
Refinement. Refinement of F^2^ against ALL reflections. The weighted R-factor wR and goodness of fit S are based on F^2^, conventional R-factors R are based on F, with F set to zero for negative F^2^. The threshold expression of F^2^ > 2sigma(F^2^) is used only for calculating R-factors(gt) etc. and is not relevant to the choice of reflections for refinement. R-factors based on F^2^ are statistically about twice as large as those based on F, and R- factors based on ALL data will be even larger.

### Fractional atomic coordinates and isotropic or equivalent isotropic displacement parameters (Å^2^)

**Table:**

	*x*	*y*	*z*	*U*_iso_*/*U*_eq_	
C1	−0.0093 (3)	0.6577 (2)	0.59295 (16)	0.0546 (7)	
H1	−0.0202	0.7107	0.6298	0.066*	
C2	−0.1114 (3)	0.6067 (3)	0.56158 (18)	0.0668 (9)	
H2	−0.1917	0.6246	0.5779	0.080*	
C3	−0.0964 (3)	0.5288 (3)	0.50594 (18)	0.0697 (9)	
H3	−0.1672	0.4959	0.4852	0.084*	
C4	0.0190 (3)	0.4993 (2)	0.48087 (16)	0.0628 (8)	
H4	0.0282	0.4472	0.4433	0.075*	
C5	0.1227 (3)	0.5493 (2)	0.51323 (14)	0.0485 (7)	
C6	0.1098 (3)	0.6296 (2)	0.56937 (14)	0.0419 (6)	
C7	0.2349 (2)	0.6687 (2)	0.58813 (13)	0.0398 (6)	
C8	0.3212 (3)	0.6114 (2)	0.54335 (12)	0.0427 (6)	
C9	0.4492 (3)	0.6300 (2)	0.54535 (14)	0.0510 (7)	
H9	0.5054	0.5918	0.5153	0.061*	
C10	0.4868 (3)	0.7095 (2)	0.59553 (14)	0.0455 (7)	
C11	0.4059 (3)	0.7687 (2)	0.64193 (13)	0.0413 (6)	
C12	0.2773 (3)	0.7488 (2)	0.63911 (12)	0.0404 (6)	
C13	0.4852 (3)	0.8433 (2)	0.68342 (14)	0.0452 (7)	
C14	0.6070 (3)	0.8217 (2)	0.66050 (15)	0.0513 (7)	
C15	0.7122 (3)	0.8755 (3)	0.68680 (18)	0.0633 (9)	
H15	0.7925	0.8582	0.6702	0.076*	
C16	0.6910 (3)	0.9561 (3)	0.73888 (18)	0.0681 (9)	
H16	0.7591	0.9949	0.7584	0.082*	
C17	0.5714 (3)	0.9816 (3)	0.76328 (16)	0.0626 (8)	
H17	0.5607	1.0376	0.7983	0.075*	
C18	0.4674 (3)	0.9256 (2)	0.73661 (14)	0.0554 (7)	
H18	0.3873	0.9425	0.7538	0.067*	
C19	0.1912 (2)	0.8073 (2)	0.69074 (13)	0.0405 (6)	
C20	0.1210 (3)	0.9003 (2)	0.66997 (15)	0.0507 (7)	
H20	0.1255	0.9270	0.6221	0.061*	
C21	0.0447 (3)	0.9532 (2)	0.72040 (17)	0.0592 (8)	
H21	−0.0021	1.0153	0.7058	0.071*	
C22	0.0362 (3)	0.9174 (3)	0.78952 (15)	0.0619 (8)	
H22	−0.0153	0.9546	0.8227	0.074*	
C23	0.1041 (3)	0.8252 (3)	0.81136 (16)	0.0708 (9)	
H23	0.0978	0.7995	0.8594	0.085*	
C24	0.1812 (3)	0.7709 (2)	0.76269 (13)	0.0562 (8)	
H24	0.2274	0.7089	0.7781	0.067*	
C25	0.3170 (3)	0.3277 (2)	0.54143 (14)	0.0493 (7)	
C26	0.4280 (3)	0.3073 (3)	0.57863 (18)	0.0666 (9)	
H26	0.5024	0.3417	0.5641	0.080*	
C27	0.4265 (4)	0.2342 (3)	0.6382 (2)	0.0808 (11)	
H27	0.5000	0.2199	0.6641	0.097*	
C28	0.3177 (5)	0.1840 (3)	0.6584 (2)	0.0906 (12)	
H28	0.3176	0.1342	0.6977	0.109*	
C29	0.2091 (4)	0.2050 (3)	0.6224 (2)	0.0904 (12)	
H29	0.1352	0.1707	0.6377	0.108*	
C30	0.2075 (3)	0.2761 (3)	0.56366 (18)	0.0686 (9)	
H30	0.1327	0.2897	0.5388	0.082*	
N1	0.2531 (2)	0.53725 (18)	0.49596 (11)	0.0497 (6)	
O1	0.2250 (2)	0.37314 (17)	0.41398 (10)	0.0763 (7)	
O2	0.4380 (2)	0.43983 (18)	0.44385 (11)	0.0795 (7)	
O3	0.60967 (18)	0.73994 (16)	0.60631 (10)	0.0573 (5)	
S1	0.31294 (8)	0.41674 (6)	0.46577 (4)	0.0583 (2)	

### Atomic displacement parameters (Å^2^)

**Table:**

	*U*^11^	*U*^22^	*U*^33^	*U*^12^	*U*^13^	*U*^23^
C1	0.0488 (19)	0.0502 (17)	0.0649 (19)	0.0022 (15)	−0.0009 (16)	0.0002 (15)
C2	0.0497 (19)	0.0610 (19)	0.090 (2)	0.0060 (17)	−0.0077 (18)	0.0020 (18)
C3	0.060 (2)	0.0595 (19)	0.089 (2)	−0.0023 (17)	−0.0197 (18)	−0.0047 (19)
C4	0.079 (2)	0.0473 (17)	0.0623 (19)	0.0004 (17)	−0.0142 (18)	−0.0081 (15)
C5	0.0597 (19)	0.0395 (15)	0.0463 (15)	0.0038 (15)	−0.0043 (14)	0.0030 (13)
C6	0.0483 (17)	0.0370 (13)	0.0404 (14)	0.0004 (13)	−0.0020 (13)	0.0078 (12)
C7	0.0504 (17)	0.0337 (14)	0.0353 (14)	0.0036 (12)	0.0024 (12)	0.0028 (12)
C8	0.0583 (18)	0.0354 (14)	0.0345 (13)	0.0007 (14)	0.0085 (14)	0.0028 (11)
C9	0.060 (2)	0.0431 (15)	0.0499 (17)	0.0039 (14)	0.0170 (15)	−0.0010 (14)
C10	0.0444 (18)	0.0460 (16)	0.0462 (15)	0.0007 (13)	0.0047 (14)	0.0045 (13)
C11	0.0469 (18)	0.0361 (13)	0.0409 (14)	0.0024 (13)	0.0024 (13)	0.0047 (12)
C12	0.0542 (18)	0.0341 (13)	0.0327 (13)	0.0069 (13)	0.0028 (12)	0.0026 (11)
C13	0.0516 (19)	0.0437 (16)	0.0404 (15)	0.0000 (14)	0.0002 (13)	0.0060 (13)
C14	0.057 (2)	0.0433 (16)	0.0539 (17)	−0.0014 (16)	−0.0010 (16)	0.0037 (14)
C15	0.053 (2)	0.060 (2)	0.076 (2)	−0.0077 (17)	−0.0024 (17)	0.0049 (18)
C16	0.069 (2)	0.057 (2)	0.079 (2)	−0.0150 (19)	−0.0148 (19)	0.0028 (18)
C17	0.080 (2)	0.0466 (17)	0.0614 (18)	−0.0068 (17)	−0.0117 (18)	−0.0040 (15)
C18	0.063 (2)	0.0449 (16)	0.0587 (17)	0.0014 (15)	−0.0005 (15)	0.0022 (15)
C19	0.0445 (16)	0.0357 (13)	0.0413 (14)	0.0019 (13)	−0.0014 (13)	−0.0010 (11)
C20	0.0557 (17)	0.0481 (16)	0.0482 (15)	0.0028 (16)	0.0013 (14)	0.0050 (14)
C21	0.0557 (18)	0.0439 (17)	0.078 (2)	0.0151 (15)	0.0026 (17)	−0.0054 (16)
C22	0.070 (2)	0.0649 (19)	0.0505 (17)	0.0154 (18)	0.0111 (15)	−0.0121 (16)
C23	0.085 (2)	0.083 (2)	0.0438 (16)	0.021 (2)	0.0115 (17)	0.0046 (16)
C24	0.068 (2)	0.0584 (18)	0.0420 (15)	0.0161 (17)	0.0077 (15)	0.0045 (13)
C25	0.0552 (18)	0.0403 (14)	0.0523 (16)	0.0042 (14)	0.0050 (16)	−0.0114 (13)
C26	0.060 (2)	0.065 (2)	0.074 (2)	0.0094 (16)	−0.0077 (18)	−0.0208 (19)
C27	0.102 (3)	0.067 (2)	0.073 (2)	0.034 (2)	−0.034 (2)	−0.019 (2)
C28	0.131 (4)	0.060 (2)	0.081 (2)	0.022 (3)	0.004 (3)	0.007 (2)
C29	0.097 (3)	0.066 (2)	0.108 (3)	0.005 (2)	0.012 (3)	0.027 (2)
C30	0.068 (2)	0.0540 (18)	0.084 (2)	0.0061 (17)	0.0014 (18)	0.0101 (17)
N1	0.0652 (16)	0.0427 (13)	0.0412 (12)	0.0012 (12)	0.0038 (11)	−0.0069 (10)
O1	0.1128 (18)	0.0644 (14)	0.0517 (12)	0.0049 (12)	−0.0123 (12)	−0.0223 (11)
O2	0.0867 (16)	0.0718 (15)	0.0801 (14)	−0.0049 (12)	0.0470 (12)	−0.0182 (12)
O3	0.0487 (12)	0.0556 (12)	0.0675 (12)	−0.0014 (11)	0.0091 (10)	−0.0024 (11)
S1	0.0794 (6)	0.0499 (4)	0.0455 (4)	0.0012 (4)	0.0136 (4)	−0.0115 (4)

### Geometric parameters (Å, º)

**Table:**

C1—C2	1.370 (4)	C16—H16	0.9300
C1—C6	1.379 (4)	C17—C18	1.381 (4)
C1—H1	0.9300	C17—H17	0.9300
C2—C3	1.383 (4)	C18—H18	0.9300
C2—H2	0.9300	C19—C24	1.386 (3)
C3—C4	1.358 (4)	C19—C20	1.388 (3)
C3—H3	0.9300	C20—C21	1.379 (4)
C4—C5	1.386 (4)	C20—H20	0.9300
C4—H4	0.9300	C21—C22	1.334 (4)
C5—C6	1.407 (4)	C21—H21	0.9300
C5—N1	1.431 (4)	C22—C23	1.373 (4)
C6—C7	1.452 (4)	C22—H22	0.9300
C7—C8	1.406 (3)	C23—C24	1.371 (4)
C7—C12	1.406 (3)	C23—H23	0.9300
C8—C9	1.381 (4)	C24—H24	0.9300
C8—N1	1.432 (3)	C25—C30	1.379 (4)
C9—C10	1.376 (4)	C25—C26	1.385 (4)
C9—H9	0.9300	C25—S1	1.740 (3)
C10—O3	1.372 (3)	C26—C27	1.392 (5)
C10—C11	1.398 (3)	C26—H26	0.9300
C11—C12	1.391 (4)	C27—C28	1.354 (5)
C11—C13	1.440 (4)	C27—H27	0.9300
C12—C19	1.487 (3)	C28—C29	1.352 (5)
C13—C14	1.386 (4)	C28—H28	0.9300
C13—C18	1.392 (4)	C29—C30	1.365 (4)
C14—C15	1.376 (4)	C29—H29	0.9300
C14—O3	1.387 (3)	C30—H30	0.9300
C15—C16	1.369 (4)	N1—S1	1.663 (2)
C15—H15	0.9300	O1—S1	1.427 (2)
C16—C17	1.383 (4)	O2—S1	1.417 (2)
			
C2—C1—C6	119.5 (3)	C16—C17—H17	119.4
C2—C1—H1	120.3	C17—C18—C13	118.4 (3)
C6—C1—H1	120.3	C17—C18—H18	120.8
C1—C2—C3	120.7 (3)	C13—C18—H18	120.8
C1—C2—H2	119.6	C24—C19—C20	117.8 (2)
C3—C2—H2	119.6	C24—C19—C12	120.0 (2)
C4—C3—C2	121.7 (3)	C20—C19—C12	122.1 (2)
C4—C3—H3	119.1	C21—C20—C19	119.9 (2)
C2—C3—H3	119.1	C21—C20—H20	120.0
C3—C4—C5	117.7 (3)	C19—C20—H20	120.0
C3—C4—H4	121.1	C22—C21—C20	121.6 (3)
C5—C4—H4	121.1	C22—C21—H21	119.2
C4—C5—C6	121.6 (3)	C20—C21—H21	119.2
C4—C5—N1	129.5 (3)	C21—C22—C23	119.6 (3)
C6—C5—N1	108.8 (2)	C21—C22—H22	120.2
C1—C6—C5	118.7 (3)	C23—C22—H22	120.2
C1—C6—C7	133.8 (2)	C24—C23—C22	120.3 (3)
C5—C6—C7	107.4 (2)	C24—C23—H23	119.9
C8—C7—C12	120.3 (2)	C22—C23—H23	119.9
C8—C7—C6	107.9 (2)	C23—C24—C19	120.8 (3)
C12—C7—C6	131.9 (2)	C23—C24—H24	119.6
C9—C8—C7	123.5 (2)	C19—C24—H24	119.6
C9—C8—N1	127.9 (2)	C30—C25—C26	120.0 (3)
C7—C8—N1	108.6 (2)	C30—C25—S1	118.9 (2)
C10—C9—C8	114.5 (2)	C26—C25—S1	121.1 (2)
C10—C9—H9	122.8	C25—C26—C27	118.8 (3)
C8—C9—H9	122.8	C25—C26—H26	120.6
O3—C10—C9	123.7 (2)	C27—C26—H26	120.6
O3—C10—C11	111.6 (2)	C28—C27—C26	119.9 (3)
C9—C10—C11	124.8 (3)	C28—C27—H27	120.1
C12—C11—C10	119.9 (2)	C26—C27—H27	120.1
C12—C11—C13	134.6 (2)	C29—C28—C27	121.2 (4)
C10—C11—C13	105.5 (2)	C29—C28—H28	119.4
C11—C12—C7	117.1 (2)	C27—C28—H28	119.4
C11—C12—C19	120.3 (2)	C28—C29—C30	120.4 (4)
C7—C12—C19	122.6 (2)	C28—C29—H29	119.8
C14—C13—C18	117.9 (3)	C30—C29—H29	119.8
C14—C13—C11	106.0 (2)	C29—C30—C25	119.7 (3)
C18—C13—C11	136.1 (3)	C29—C30—H30	120.1
C15—C14—C13	124.7 (3)	C25—C30—H30	120.1
C15—C14—O3	123.9 (3)	C5—N1—C8	107.3 (2)
C13—C14—O3	111.3 (3)	C5—N1—S1	122.09 (19)
C16—C15—C14	115.7 (3)	C8—N1—S1	122.47 (18)
C16—C15—H15	122.1	C10—O3—C14	105.5 (2)
C14—C15—H15	122.1	O2—S1—O1	119.98 (13)
C15—C16—C17	121.9 (3)	O2—S1—N1	106.63 (13)
C15—C16—H16	119.0	O1—S1—N1	106.34 (13)
C17—C16—H16	119.0	O2—S1—C25	108.57 (14)
C18—C17—C16	121.3 (3)	O1—S1—C25	108.66 (13)
C18—C17—H17	119.4	N1—S1—C25	105.76 (11)
			
C6—C1—C2—C3	−0.9 (4)	C16—C17—C18—C13	−0.9 (4)
C1—C2—C3—C4	0.6 (5)	C14—C13—C18—C17	0.4 (4)
C2—C3—C4—C5	0.4 (4)	C11—C13—C18—C17	−179.1 (3)
C3—C4—C5—C6	−1.1 (4)	C11—C12—C19—C24	−77.7 (3)
C3—C4—C5—N1	−177.3 (3)	C7—C12—C19—C24	99.5 (3)
C2—C1—C6—C5	0.2 (4)	C11—C12—C19—C20	100.7 (3)
C2—C1—C6—C7	177.5 (3)	C7—C12—C19—C20	−82.1 (3)
C4—C5—C6—C1	0.8 (4)	C24—C19—C20—C21	0.0 (4)
N1—C5—C6—C1	177.7 (2)	C12—C19—C20—C21	−178.5 (2)
C4—C5—C6—C7	−177.2 (2)	C19—C20—C21—C22	0.2 (4)
N1—C5—C6—C7	−0.2 (3)	C20—C21—C22—C23	−0.5 (5)
C1—C6—C7—C8	−177.7 (3)	C21—C22—C23—C24	0.7 (5)
C5—C6—C7—C8	−0.2 (3)	C22—C23—C24—C19	−0.6 (5)
C1—C6—C7—C12	1.5 (5)	C20—C19—C24—C23	0.2 (4)
C5—C6—C7—C12	179.0 (2)	C12—C19—C24—C23	178.7 (3)
C12—C7—C8—C9	−0.9 (4)	C30—C25—C26—C27	0.0 (4)
C6—C7—C8—C9	178.4 (2)	S1—C25—C26—C27	−179.2 (2)
C12—C7—C8—N1	−178.8 (2)	C25—C26—C27—C28	0.5 (5)
C6—C7—C8—N1	0.5 (3)	C26—C27—C28—C29	−1.2 (5)
C7—C8—C9—C10	0.4 (4)	C27—C28—C29—C30	1.2 (6)
N1—C8—C9—C10	177.9 (2)	C28—C29—C30—C25	−0.6 (5)
C8—C9—C10—O3	179.7 (2)	C26—C25—C30—C29	0.0 (4)
C8—C9—C10—C11	0.1 (4)	S1—C25—C30—C29	179.2 (2)
O3—C10—C11—C12	−179.8 (2)	C4—C5—N1—C8	177.2 (3)
C9—C10—C11—C12	−0.1 (4)	C6—C5—N1—C8	0.6 (3)
O3—C10—C11—C13	1.3 (3)	C4—C5—N1—S1	−33.7 (4)
C9—C10—C11—C13	−179.0 (2)	C6—C5—N1—S1	149.69 (18)
C10—C11—C12—C7	−0.4 (3)	C9—C8—N1—C5	−178.4 (2)
C13—C11—C12—C7	178.1 (2)	C7—C8—N1—C5	−0.7 (3)
C10—C11—C12—C19	177.0 (2)	C9—C8—N1—S1	32.6 (3)
C13—C11—C12—C19	−4.5 (4)	C7—C8—N1—S1	−149.66 (18)
C8—C7—C12—C11	0.9 (3)	C9—C10—O3—C14	179.7 (2)
C6—C7—C12—C11	−178.3 (2)	C11—C10—O3—C14	−0.7 (3)
C8—C7—C12—C19	−176.5 (2)	C15—C14—O3—C10	−179.2 (3)
C6—C7—C12—C19	4.4 (4)	C13—C14—O3—C10	−0.3 (3)
C12—C11—C13—C14	179.9 (3)	C5—N1—S1—O2	171.21 (19)
C10—C11—C13—C14	−1.4 (3)	C8—N1—S1—O2	−44.3 (2)
C12—C11—C13—C18	−0.5 (5)	C5—N1—S1—O1	42.1 (2)
C10—C11—C13—C18	178.1 (3)	C8—N1—S1—O1	−173.43 (19)
C18—C13—C14—C15	0.4 (4)	C5—N1—S1—C25	−73.3 (2)
C11—C13—C14—C15	180.0 (3)	C8—N1—S1—C25	71.2 (2)
C18—C13—C14—O3	−178.6 (2)	C30—C25—S1—O2	−167.4 (2)
C11—C13—C14—O3	1.1 (3)	C26—C25—S1—O2	11.8 (3)
C13—C14—C15—C16	−0.5 (4)	C30—C25—S1—O1	−35.4 (3)
O3—C14—C15—C16	178.3 (2)	C26—C25—S1—O1	143.8 (2)
C14—C15—C16—C17	−0.1 (5)	C30—C25—S1—N1	78.5 (2)
C15—C16—C17—C18	0.8 (5)	C26—C25—S1—N1	−102.3 (2)

### Hydrogen-bond geometry (Å, º)

*Cg*3 and *Cg*4 are the centroids of rings C1–C6 and C7–C12, respectively.

**Table:**

*D*—H···*A*	*D*—H	H···*A*	*D*···*A*	*D*—H···*A*
C4—H4···O1	0.93	2.34	2.924 (4)	121
C9—H9···O2	0.93	2.34	2.926 (3)	121
C2—H2···O3^i^	0.93	2.57	3.464 (4)	160
C17—H17···*Cg*4^ii^	0.93	2.81	3.683 (3)	156
C22—H22···*Cg*3^iii^	0.93	2.95	3.722 (3)	141

Symmetry codes: (i) *x*−1, *y*, *z*; (ii) −*x*+1, *y*+1/2, −*z*+3/2; (iii) −*x*, *y*+1/2, −*z*+3/2.
